# Seasonal modification of the association between temperature and adult emergency department visits for asthma: a case-crossover study

**DOI:** 10.1186/1476-069X-11-55

**Published:** 2012-08-16

**Authors:** Jessie P Buckley, David B Richardson

**Affiliations:** 1 Department of Epidemiology, University of North Carolina at Chapel Hill, McGavran-Greenberg Hall, CB #7435, Chapel Hill, NC 27599-7435, USA

**Keywords:** Asthma, Temperature, Season, Case-crossover

## Abstract

**Background:**

The objective of this study is to characterize the effect of temperature on emergency department visits for asthma and modification of this association by season. This association is of interest in its own right, and also important to understand because temperature may be an important confounder in analyses of associations between other environmental exposures and asthma. For example, the case-crossover study design is commonly used to investigate associations between air pollution and respiratory outcomes, such as asthma. This approach controls for confounding by month and season by design, and permits adjustment for potential confounding by temperature through regression modeling. However, such models may fail to adequately control for confounding if temperature effects are seasonal, since case-crossover analyses rarely account for interactions between matching factors (such as calendar month) and temperature.

**Methods:**

We conducted a case-crossover study to determine whether the association between temperature and emergency department visits for asthma varies by season or month. Asthma emergency department visits among North Carolina adults during 2007–2008 were identified using a statewide surveillance system. Marginal as well as season- and month-specific associations between asthma visits and temperature were estimated with conditional logistic regression.

**Results:**

The association between temperature and adult emergency department visits for asthma is near null when the overall association is examined [odds ratio (OR) per 5 degrees Celsius = 1.01, 95% confidence interval (CI): 1.00, 1.02]. However, significant variation in temperature-asthma associations was observed by season (chi-square = 18.94, 3 degrees of freedom, p <0.001) and by month of the year (chi-square = 45.46, 11 degrees of freedom, p <0.001). ORs per 5 degrees Celsius were increased in February (OR = 1.06, 95% CI: 1.02, 1.10), July (OR = 1.16, 95% CI: 1.04, 1.29), and December (OR = 1.04, 95% CI: 1.01, 1.07) and decreased in September (OR = 0.92, 95% CI: 0.87, 0.97).

**Conclusions:**

Our empirical example suggests that there is significant seasonal variation in temperature-asthma associations. Epidemiological studies rarely account for interactions between ambient temperature and temporal matching factors (such as month of year) in the case-crossover design. These findings suggest that greater attention should be given to seasonal modification of associations between temperature and respiratory outcomes in case-crossover analyses of other environmental asthma triggers.

## Background

Ambient temperature is a potential confounding factor in many studies of the health effects of environmental exposures, such as air pollution. Time-series and case-crossover designs have become widely used in epidemiological studies of environmental triggers of respiratory health outcomes, such as asthma. The case-crossover study design with time-stratified sampling is appealing since it affords control for confounding by day of week, month, and season of year by design [1,2]. Control of time-varying confounders, such as daily variation in ambient temperature, may be achieved through regression modeling. Some studies have modeled temperature effects using simple linear, or linear-quadratic functions of temperature [3,4]; others have approached modeling of temperature as a nuisance factor by using more flexible spline functions and incorporation of latency periods [5,6]. However, such models may fail to adequately control for confounding by temperature if the effects of temperature on respiratory outcomes operate through seasonal pathways.

**Figure 1:**
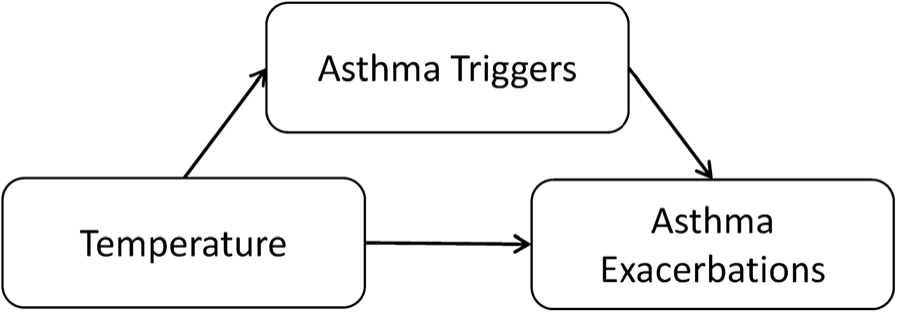
Directed acyclic graph for the total effect of temperature on asthma exacerbations.

Asthma exacerbation is an outcome for which seasonal variation in temperature effects may be particularly important. The total effect of temperature on asthma exacerbations includes both direct effects and indirect effects. Figure 1 is a simplified directed acyclic graph illustrating associations between temperature and asthma exacerbations. As indicated by the directed edge between temperature and asthma exacerbations, temperature may have direct effects on inflammation pathways or airway hyper-responsiveness causing asthma exacerbations. In addition, temperature may have indirect effects on asthma exacerbation that operate through other asthma triggers (e.g. viral infections, pollen, air pollution, mold, house dust mites, cockroaches, heating and air conditioning use, time spent outdoors, and activity levels). These indirect effects are represented in the causal graph by the directed edge between temperature and asthma triggers, and between asthma triggers and asthma exacerbations. In the current paper we focus on estimation of the total effect of temperature on asthma exacerbation, which includes both the direct and indirect pathways. As indicated in Figure 1, our proposed causal structure implies that adjustment for asthma triggers such as air pollution is not appropriate in this analysis since air pollutants are affected by temperature. Consequently, air pollution is a causal intermediate and part of the total effect of temperature on asthma, not a confounder.

The association between temperature and asthma exacerbations is of interest in its own right, and also important to understand because temperature may be an important confounder in analyses of associations between other asthma triggers and asthma (Figure 1). For example, the case-crossover study design is commonly used to investigate associations between air pollution and respiratory outcomes, such as asthma. This approach controls for confounding by month and season by design, and permits adjustment for potential confounding by temperature through regression modeling. However, if there are seasonal differences in either direct or indirect effects of temperature on asthma, failure to account for this heterogeneity may result in poor control for confounding in studies of air pollution and other environmental risk factors. To-date, epidemiological studies have rarely accounted for interactions between temporal matching factors (such as month of year) in the case-crossover design and this potential confounder, ambient temperature.

In this paper we use empirical data for emergency department visits for asthma among North Carolina adults to characterize the total effect of temperature on emergency department visits for asthma and modification of this association by season. Using these data, we illustrate the importance of considering variation in temperature-respiratory health outcome associations by season of year and by finer stratification into month of year.

## Methods

### Study population

The study population was defined as all residents of North Carolina greater than 18 years of age during the period 1/1/2007-12/31/2008. North Carolina emergency departments are mandated to submit select data elements to the North Carolina Disease Event Tracking and Epidemiologic Collection Tool (NC DETECT) for public health purposes. We used NC DETECT to ascertain the age, sex, county of residence, and date of emergency department visits with a primary diagnosis of asthma (ICD-9-CM code: 493.xx).

### Temperature data

Temperature data obtained from the State Climate Office of North Carolina were collected from two sources: first order stations and cooperative observation stations. First order stations are automated, have quality control procedures, and collect hourly data on ambient temperature. Cooperative observation station data are gathered by trained volunteers who record daily minimum and maximum temperatures. Daily ambient temperatures for each station were calculated as the mean of the hourly observations (first order stations) or the average of the minimum and maximum recorded value (cooperative observation stations). Two stations were excluded due to missing or implausible values. For counties with more than one weather station (N = 35), values were averaged over all stations to obtain the daily average temperature for that county. Ambient temperatures were available for 94 of the 100 counties in North Carolina.

### Study design and data analysis

The total effect of temperature on asthma emergency department visits was assessed using a case-crossover study design. Cases were matched to themselves and daily temperature on the day of the event was compared to exposure on control days. This design adjusts for time-fixed confounders by matching within person. Using a time-stratified bidirectional approach for referent selection, control days were defined as all days in the same calendar month as the asthma visit, matched on day of the week (3 to 4 control periods per case). This control period selection approach adjusts for confounding by day of the week, month of the year, and season by matching. It also reduces long-term trends in other time varying confounders by restricting case to control period contrasts to the one month referent window [1,7]. Variation in asthma triggers during the one month referent window is more likely due to an effect of temperature than vice versa, since most asthma triggers do not affect temperature on this time scale (Figure 1). For example, temperature affects the release of pollen into the environment, which in turn affects asthma exacerbation (pollen does not affect temperature). This causal structure holds for many unmeasured environmental causes of asthma emergency department visits. Thus, the total effect of temperature includes pathways through other asthma triggers and adjustment for these variables is inappropriate.

Case periods were assigned the average temperature of their county of residence on the day of the emergency department visit and control periods were assigned the temperature on the matched control days. Lagged exposure variables were constructed to represent the temperatures on each of the 6 days prior to the event (lags 1–6). Cases that occurred on days with missing ambient temperature (N = 2,973) did not differ from the total case group in the distribution of measured covariates.

Odds ratios (OR) and 95% confidence intervals (CI) for associations between temperature and asthma emergency department visits were estimated using conditional logistic regression (SAS Version 9.2, Cary, North Carolina). Nonlinearity was evaluated by entering higher order polynomial terms for temperature in the model and conducting likelihood ratio tests. Odds ratio modification was assessed via likelihood ratio tests comparing models with and without an interaction term between temperature and a nominal variable for season or month. Seasons were defined using the following three month periods: winter (December – February), spring (March – May), summer (June – August), and fall (September – November). The goodness of model fit was compared for lagged exposures (lags 1–6) to identify the lag assumption that maximized the log likelihood. Analyses were adjusted for a binary indicator of North Carolina state holidays since they are time-varying but were not matched on.

This study utilized deidentified data and was therefore exempt from Institutional Review Board review.

## Results

Characteristics of the 53,156 emergency department visits for asthma during the study period are reported in Table 1. The number of asthma visits peaked in February and was lowest in July. Mean daily temperatures (°C) were coldest in January (mean 5.8, SD 6.1) and February (mean 6.4, SD 5.2) and warmest in July (mean 24.7, SD 2.4) and August (mean 25.7, SD 2.9).

**Table 1:** Characteristics of adult emergency department visits for asthma in North Carolina, 2007-2008

**Characteristic**		**Number of Visits**	**%**
Age (years)	19-24	8,295	15.6
	25-44	23,849	44.9
	45-64	15,332	28.8
	≥65	5,680	10.7
Sex	Female	35,397	66.6
	Male	17,756	33.4
	Missing	3	0.0
Holiday	Yes	1,549	2.9
	No	51,607	97.1
Year	2007	26,406	49.7
	2008	26,750	50.3
Month	January	4,527	8.5
	February	4,864	9.2
	March	4,476	8.4
	April	4,523	8.5
	May	4,592	8.6
	June	3,997	7.5
	July	3,675	6.9
	August	4,087	7.7
	September	4,650	8.8
	October	4,662	8.8
	November	4,550	8.6
	December	4,553	8.6

**Figure 2:**
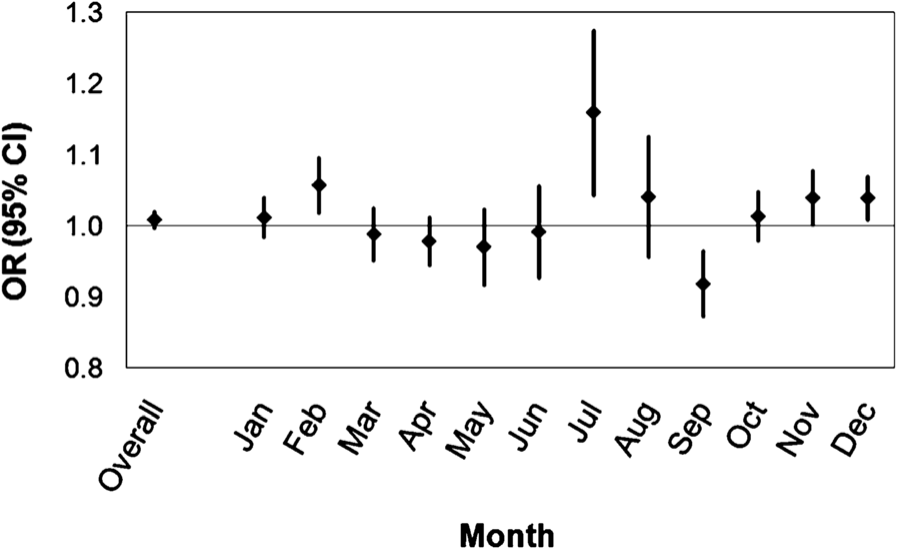
Odds ratios for adult emergency department visits for asthma per 5°C by month.

We first estimated the marginal association. The OR for the association between asthma emergency department visits and each 5°C increase in temperature on the day of the visit (lag 0) was 1.01 (95% CI: 1.00, 1.02). Next, we assessed whether this association was modified by season by including product terms to allow the temperature (lag 0) association to vary by season (Table 2). There was strong evidence of variation in the estimated association by season (chi-square = 18.94, 3 degrees of freedom, p <0.001). Finer stratification by month of the year also suggested strong evidence of effect measure modification (chi-square = 45.46, 11 degrees of freedom, p <0.001) as illustrated in Figure 2. The magnitude and direction of association were similar at 0-, 1-, and 2-day lagged temperatures, with model goodness of fit slightly better at a 0-day lag for season and a 1-day lag for month (see Additional File 1).

**Table 2:** Odds ratios for adult emergency department visits for asthma per 5°C by season

	**Mean Daily Average Ambient Temperature (°C)**	**Odds Ratio**^**a **^**(95% Confidence Interval)**	**Test for heterogeneity( *χ* **^**2 **^**, DF, p-value)**
Overall	15.9	1.01 (1.00, 1.02)	
Season			18.94, 3, p<0.001
Winter	6.9	1.03 (1.01, 1.05)	
Spring	15.3	0.97 (0.95, 0.99)	
Summer	25.0	1.04 (0.99, 1.09)	
Fall	16.0	1.01 (0.98, 1.03)	

^a^Odds ratio per 5°C increase in temperature adjusted for holidays, based on log-linear conditional logistic regression models fitted separately for each season.

The estimated association between temperature and asthma visits varied over the year, with positive linear associations in the winter and summer and a negative association in the spring. A quadratic term for temperature was significant in the fall, with lower and higher temperatures associated with decreased odds of an asthma visit. This seasonal pattern was also evident in the month-specific analyses, which demonstrated notable variation by calendar month. The odds of an asthma emergency department visit per 5°C increase in temperature on the day of the event were significantly increased in February (OR = 1.06, 95% CI: 1.02, 1.10), July (OR = 1.16, 95% CI: 1.04, 1.29), and December (OR = 1.04, 95% CI: 1.01, 1.07) and decreased in September (OR per 5°C = 0.92, 95% CI: 0.87, 0.97).

## Discussion

The total effect of daily average temperature on emergency department visits for asthma in North Carolina exhibited heterogeneity by season or month; higher temperatures on the day of the event than on control days were associated with increased risk of emergency department visits during winter and summer months and decreased risk in the spring and September. This heterogeneity may be due to differences in absolute temperature over the year but likely also reflects seasonal variation in the prevalence of other asthma risk factors that interact with or mediate the effect of temperature on exacerbation. Many time-varying risk factors for asthma are affected by temperature, including viral infections, pollen, air pollution, mold, house dust mites, cockroaches, heating and air conditioning use, time spent outdoors, and activity levels.

Most prior studies of the association between temperature and emergency department or hospital visits for asthma have reported inverse associations [8-15], although null [16-19] or positive [20] relations have also been described. Few assessments of the association between temperature and asthma exacerbations have considered effect modification by season. A case-crossover study of temperature and childhood asthma emergency department visits in Ottawa, Canada examined 6-hour changes in ambient temperature or relative humidity and reported no seasonal modification [21]. A study in Oulu, Finland reported that the direction of the correlation between daily emergency department visits for asthma and ambient temperature was opposite in the summer and winter, though neither variable was significantly correlated with asthma visits [15]. Temperature modified the association between elemental carbon and pediatric emergency department visits for asthma in Saint Louis, Missouri, increasing risk in the summer and fall and decreasing risk in the spring [22]. Because temperature affects many asthma risk factors that vary in prevalence and seasonality by region, patterns of association between temperature and asthma are also expected to differ geographically.

Time-varying factors other than temperature were not ascertained. Case-crossover studies suffer from confounding when the baseline risk of the outcome is not constant within the referent window [23]. The time-stratified, bidirectional approach to control period sampling within a one-month referent window constrains variation in potential time-varying confounders but does not eliminate it. While long-term trends are controlled by matching within the referent window, it is possible that short-term trends in other asthma triggers could have biased the observed associations between temperature and asthma visits. However, because many time-varying risk factors for asthma are intermediates on the causal pathway (e.g., aeroallergens, air pollution, activity patterns), associations estimated in this study represent the total effect of temperature on asthma risk. Estimating the direct effect of temperature on asthma emergency department visits is not the objective of this analysis and would require statistical adjustment for each of these intermediate variables, which can result in over-adjustment bias and is inappropriate if the mediator is an effect measure modifier [24,25]. The results of this analysis may be biased, however, if there are time-varying factors associated with asthma risk that are not affected by temperature but have a common cause (e.g., ozone levels and temperature are both affected by sunlight and both affect asthma risk).

## Conclusions

In the current study we illustrate seasonal differences in the direction of association with the purpose of emphasizing that averaging over the entire calendar year may not appropriately represent the complexity of the effect of temperature on risk of asthma exacerbation. Seasonal or monthly variation in the association between temperature and asthma may be due to a direct effect of temperature, which varies over the year as a function of absolute temperature, or to indirect effects of temperature on other asthma risk factors that vary seasonally and interact with or mediate the effect of temperature on exacerbation. These environmental and behavioral asthma triggers are often difficult to measure (e.g., viral infections, indoor air pollution, time spent outdoors), particularly in studies using monitoring data to assign exposures.

Therefore, to avoid residual confounding, case-crossover studies of environmental exposures should consider modification of temperature-asthma associations by temporal matching factors such as month or season. Accounting for this heterogeneity is particularly important when estimating small effect sizes typical of environmental epidemiology studies.

## Abbreviations

C: Celsius; CI: Confidence interval; NC DETECT: North Carolina Disease Event Tracking and Epidemiologic Collection Tool; OR: Odds ratio.

## Competing interests

The authors declare that they have no competing interests.

## Authors’ contributions

DR conceived of and designed the study, acquired data, and provided critical review of content. JB conducted data analysis, interpreted results, and drafted the manuscript. Both authors read and approved the final manuscript.

## Supplementary Material

Additional file 1 ** Mean daily average ambient temperature and odds ratios for adult emergency department visits for asthma per 5°C by season, month, and lag in North Carolina, 2007–2008. **This table provides additional estimates of association between adult emergency department visits for asthma and temperature by season, month, and lag. Click here for file
